# A POCS super resolution restoration algorithm based on BM3D

**DOI:** 10.1038/s41598-017-15273-0

**Published:** 2017-11-08

**Authors:** Jian Chen, WeiGuo Wang, TingXia Liu, ZhenDong Zhang, HuiBin Gao

**Affiliations:** 1 Changchun Institute of Optics Fine Mechanics and Physics, Chinese Academy of Sciences, Changchun, 130033 China; 2 University of Chinese Academy of Sciences, Beijing, 100049 China; 3 College of Communication Engineering, Jilin University, Changchun, 130012 China

## Abstract

The inherent shortcoming of POCS (Projection Onto Convex Sets) is its sensitiveness to noise. The restoration quality of POCS based super resolution will severely decline when the noise is larger. In practical applications, the low resolution images generally include some kinds of noise, such as camera internal noise, transmission system noise and coherent noise. Therefore POCS cannot be used directly in super-resolution restoration for observed low resolution images. In order to solve the noise sensitive problem of the traditional POCS restoration algorithm, we firstly propose to optimize the BM3D (Block-Matching 3D) filtering by mean pre-screening of image blocks and limiting the number of image blocks. Then we combine the optimized BM3D filtering with POCS restoration in this paper. Experimental results show that the proposed POCS super resolution restoration algorithm based on BM3D can achieve better restoration effect when the low resolution images contain noise. Furthermore no noise can be perceived in the restored high resolution image basically.

## Introduction

Projection Onto Convex Sets (POCS) is intuitive and simple for code implementation. The algorithm of POCS uses prior knowledge set intersection to search for the solution space, which has inherent advantages on the introduction of prior knowledge. Meanwhile it can build complex image degradation model. Therefore POCS becomes one of most popular spatial super resolution restoration methods. For instance, POCS is used for super resolution restoration to the compressed video data in^1^.

However, POCS has its inherent shortcoming, which is extremely sensitive to noise. The restoration quality of using POCS will severely decline due to larger noise. In practical applications, the obtained low resolution images usually contain various noises such as internal noise of infrared camera, noise in transmission system and coherent noise, which make POCS not be applied directly to super resolution restoration.

The appearance of non local filtering is late, but the development speed of it is very fast. It is one of the most attractive directions in the current image filtering theories. The algorithm assumes that there are many similar image blocks in the image, which contain similar useful image information and the noise can be filtered out by weighted average. This method is called non local filtering because the selection of image blocks is not limited to the neighborhoods^2,3^. The simplest non local filtering method is NLM (Nonlocal Means), while BM3D is one of the best filtering methods at present^4^.

Considering the case of noise interference, this paper proposes a POCS super resolution restoration algorithm based on BM3D, which can effectively depress noise and get better restoration effect even for low resolution images with low SNR (Signal to Noise Ratio).

## POCS super resolution restoration based on BM3D (BPOCS)

### Algorithm introduction

The super resolution restoration using POCS is convenient to bring into prior knowledge and build complex image degradation model, which is a more mature method widely used in many occasions^5,6^. However, this method is sensitive to noise and the restoration quality will decline dramatically for image sequence with low SNR^7,8^. Considering the good denoising performance and the capability of preserving edge details, we consider combining BM3D and POCS, in which the image sequence with high SNR are obtained from the low resolution image sequence using BM3D filtering^9–11^.

**Figure 1:**
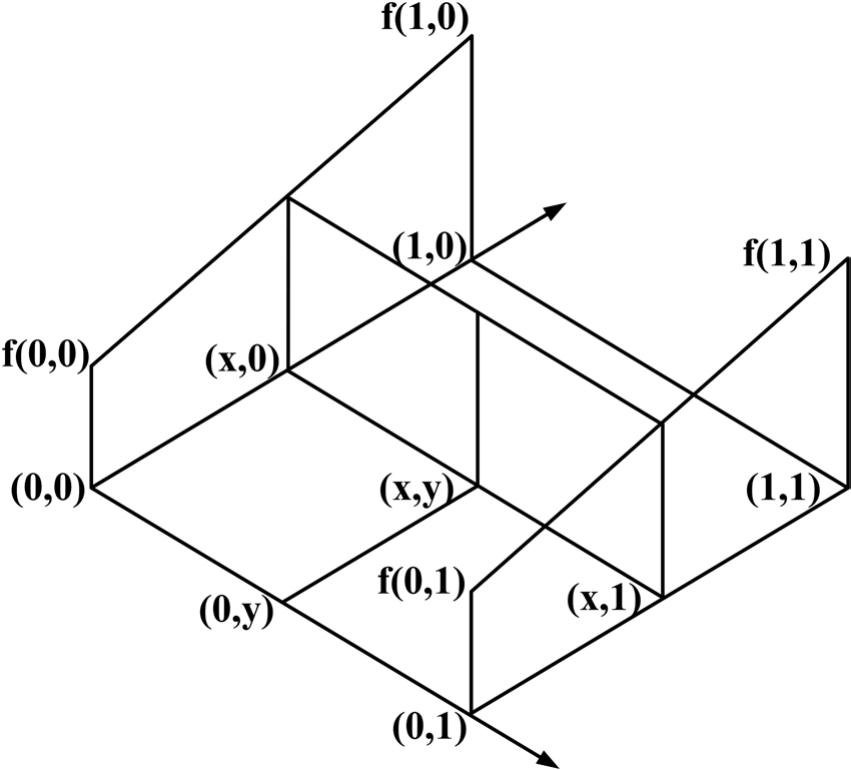
Diagrammatic sketch of bilinear interpolation.

Then the super resolution restoration using POCS is performed. The original estimation of the high resolution image is obtained from the low resolution image reference frame using interpolation. The common interpolation methods are nearest neighbor interpolation, bilinear interpolation and high order interpolation. The computation of nearest neighbor interpolation is simple. However, the interpolation results are prone to aliasing. The effect of bilinear interpolation is better than that of the nearest neighbor interpolation, but it will smooth the edge. The computation complexity of high order interpolation is high. Considering the large amount of computation and interpolation effect, the bilinear interpolation method is introduced to construct the reference frame in this paper, as shown in Fig. 1.

As shown in Fig. 1, suppose the four neighborhood points are $f(0,0)$,$f(0,1)$,$f(1,0)$ and $f(1,1)$. The realization process of the bilinear interpolation is described as follows.

Step1: The first order bilinear interpolation is used in the direction of x to get $f(x,0)$:

1 $$f(x,0)=f(0,0)+x[f(1,0)\,-\,f(0,0)]$$

Step2: The first order bilinear interpolation is used again in the direction of x to get $f(x,1)$:

2 $$f(x,1)=f(0,1)+x[f(1,1)\,-\,f(0,1)]$$

Step3: The first order bilinear interpolation is used in the direction of y to get $f(x,y)$:

3 $$f(x,y)=f(x,0)+y[f(x,1)\,-\,f(x,0)]$$

From the above, the bilinear interpolation to get $f(x,y)$ is:

4 $$f(x,y)=[f(1,0)\,-\,f(0,0)]x+[f(0,1)\,-\,f(0,0)]y+[f(1,1)+f(0,0)\,-\,f(0,1)\,-\,f(1,0)]xy+f(0,0)$$

The high resolution image $\hat{H{p}_{r}}$ can be obtained after bilinear interpolation using equation (4). Similarly, the other low resolution images $\{L{p}_{i}\}$ $i\ne r$ take the same interpolation operation to obtain the corresponding high resolution images $\{\hat{H{p}_{i}}\}$. For every $\hat{H{p}_{i}}$, the image block $Patc{h}_{i,M}$ of size $M\times M$ is taken with $\hat{H{p}_{i}}(x,y)$ as the centre. The image block $Patc{h}_{r,L}$ $L > M$ of size $L\times L$ is taken from the reference frame $\hat{H{p}_{r}}$ with the corresponding central position $\hat{H{p}_{r}}(x,y)$. A series of small image blocks $Patc{h}_{r,M}^{h,v}$ is taken by sliding pixel by pixel in the large image block $Patc{h}_{r,L}$. Where $h$ in $Patc{h}_{r,M}^{h,v}$ represents the horizontal displacement of the small image block corresponding to the centre (*x*,*y*) of $Patc{h}_{r,L}$, which is $-\frac{L}{2}\le h\le \frac{L}{2}$, and *v* in $Patc{h}_{r,M}^{h,v}$ represents the vertical displacement of the small image block corresponding to the centre (*x*,*y*) of $Patc{h}_{r,L}$, which is $-\frac{L}{2}\le v\le \frac{L}{2}$. The mean square error between $Patc{h}_{i,M}$ and $Patc{h}_{r,M}^{h,v}$ is calculated by:

5 $$MSE(h,v)=\frac{1}{M\times M}\sum _{m=0}^{M-1}\sum _{n=0}^{M-1}{[Patc{h}_{i,M}(m,n)-Patc{h}_{r,M}^{h,v}(m,n)]}^{2}$$

The position with the least mean square error corresponding to $(h,v)$ is the relative displacement of the two frames. Therefore the motion parameter estimation with the sub pixel level can be obtained.

According to the model, the point spread function $h$ and its range of action between the two frames is estimated. Then $\hat{H{p}_{i}}(x+h,y+v)$ $i\ne r$ is mapped to the coordinate of the reference frame $\hat{H{p}_{r}}(x,y)$ and its neighborhood according to the displacement relationship of the two frames. The residual error is calculated by:

6 $$\varepsilon =| \hat{H{p}_{r}}(x,y)\,-\,h\times \hat{H{p}_{i}}(x+h,y+v)| $$

It is necessary to correct $\hat{H{p}_{r}}(x,y)$ if the residual is greater than the predefined threshold *T*. This correction is a process of iteration, because the influence range of reference frame pixel points is superimposed, and the result of one correction may cause the value of the last correction to be greater than the error threshold.

All pixel points are corrected after iteration. The high resolution image *Hp* is finally estimated.

In conclusion, the steps of POCS super resolution restoration based on BM3D are described as follows.

Step1: The image sequence with higher SNR is obtained from low SNR images using BM3D filtering.

Step2: The reference frame of the restoration image is obtained from one degraded image after bilinear interpolation.

Step3: For the reference frame, the sub-pixel motion estimation can be got for every low resolution images in the sequence.

Step4: The low resolution image mapping to high resolution grid correct reference frame based on motion estimation and system PSF.

Step5: Repeat Step4 until the error is less than the given threshold.

**Figure 2:**
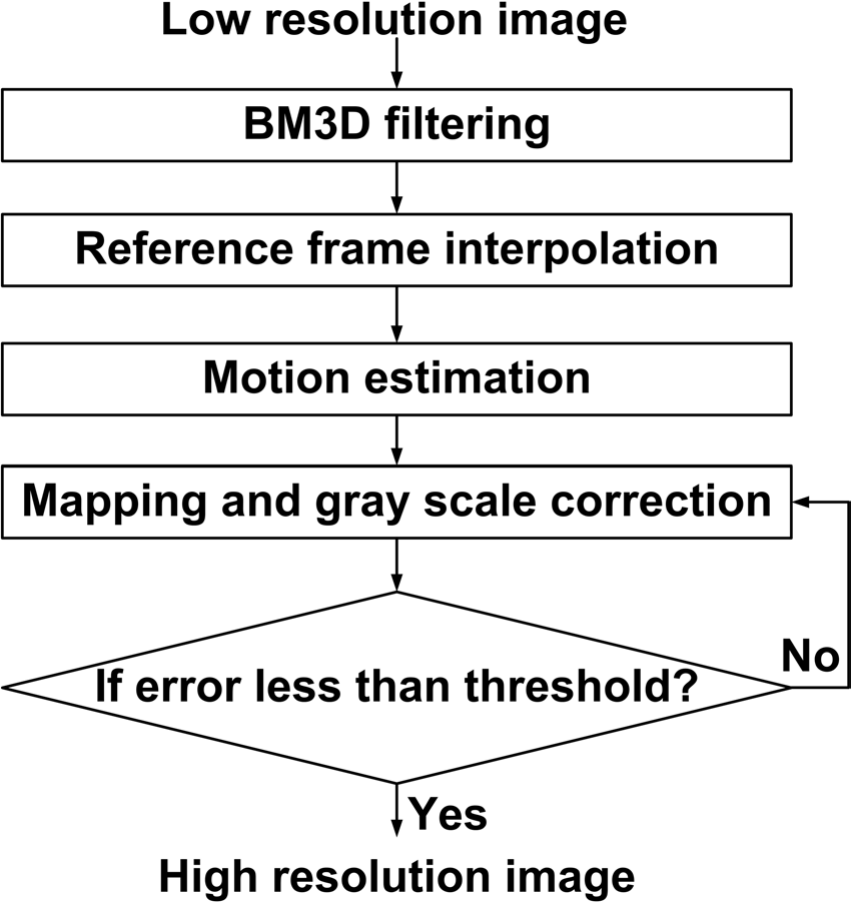
Flow chart of POCS super resolution restoration based on BM3D.

The flow chart of POCS super resolution restoration based on BM3D (BPOCS) is shown in Fig. 2.

The process of BM3D filtering includes grouping, transform domain filtering and aggregating. Therefore, its computational complexity is high. Meanwhile, as an iterative algorithm, POCS is also time consuming. In order to improve the implementation efficiency and guarantee the restoration quality of super resolution restoration, we optimize the algorithm from two aspects: the block pre-selection of BM3D and the parameter selection of POCS.

### Block pre-selection optimization of BM3D

For the large computation of the BM3D filtering method, we propose the method of mean pre-screened image block and limiting the number of packet image blocks to reduce the computation of BM3D method. The improved algorithm can guarantee the performance of filtering and greatly reduce the running time.

In BM3D algorithm, most time is consumed in the step of grouping. According to the theoretical analysis, BM3D algorithm needs to find similar image block in the entire low resolution image. Assuming the size of the low resolution image is *N* × *N* and the size of image block is *r* × *r*, the computational cost of finding a similar image block is ${r}^{2}\times {N}^{2}\times {N}^{2}$ when grouping, which is a very time-consuming process. For example, the computational time of BM3D filtering is 0.6 s for the low resolution Lena image with size 128 × 128. Therefore we consider simplifying the progress of grouping and propose a fast method to pre-select the similar image blocks.

Two image blocks are considered to have the similar gray values if they are similar both in the whole and in the details. In other words two image blocks are not similar if they have different gray values. The Euclidean distance is large if the difference of two image blocks gray is large. The gray mean value is firstly calculated to eliminate the image blocks which Euclidean distance is large. The mean value *m*_*R*_ and *m*_*C*_ of the reference image block *Z*_*R*_ and matching image block *Z*_*c*_ are calculated to satisfy the condition below shown as equation (7).

7 $$1\,-\,{\tau }_{m} < \frac{{m}_{R}}{{m}_{C}} < 1+{\tau }_{m}$$

The condition in equation (7) indicates that the mean gray values of the two image blocks are similar. Then the Euclidean distance of two image blocks is calculated to determine the similarity. In equation (7) ${\tau }_{m}$ is the predefined threshold. The calculation of mean gray value is simple. Therefore the process of grouping is greatly accelerated.

The search for similar image blocks can be limited to a certain range such as the neighborhood *D* × *D* of the current pixel. It will not affect the precision of filtering and aggregating. At the same time, the number of blocks in every group can be properly decreased by using the completeness of three-dimensional array. For example, if the threshold is defined as ${\tau }_{N}$, the calculation will stop when the number of similar image block achieves ${\tau }_{N}$ in every group, which further simplifies the process of grouping.

### Parameter optimization of POCS

For the long iteration of the POCS super resolution restoration algorithm, we propose parameter optimization of POCS. The improved algorithm can guarantee the performance of the super resolution restoration and greatly reduce the running time.

#### Selection of confidence interval σ_v_

The priori border of noise convex set is determined by noise confidence interval *σ*_*v*_. The bigger the border of convex set is, the larger the *σ*_*v*_ is, and the bigger the solution space is bigger. Therefore, it is more difficult to locate a more accurate solution. Although noise can be effectively suppressed, the quality of the restored image will be low. If *σ*_*v*_ is smaller, the number of points with gray correction is increasing, which is beneficial to maintain the edge details. Meanwhile it will also retain more noise. Considering that BM3D has been used to filter previously, the noise has been effectively suppressed. Therefore *σ*_*v*_ is selected as a smaller value, which can guarantee the restoration quality of high resolution image.

#### The relationship between resolution of the restored image and the number reference frames

Assuming the resolution of low resolution image $M\times N$, the vector $Lp={\{L{{p}_{1}}^{T},L{{p}_{2}}^{T},{\rm{...}},L{{p}_{n}}^{T}\}}^{T}$ is defined to represent the column vector $nMN\times 1$ which is composed by low resolution images. If assuming the size of the restored high resolution image is supposed to be $r\times M\times N$, where *r* is magnification times, the imaging model is given by:

8 $$Lp=HX+N$$

In equation (8), *H* is the system degradation matrix with dimension $nMN\times {r}^{2}MN$ which includes a complex imaging process such as down sampling, target motion, blurring and defocusing. Both $Lp$ and *N* are vector with dimension $nMN\times 1$. The equation (8) is a linear equation set, which has no solution when $n < {r}^{2}$ and has approximate solution when $n\ge {r}^{2}$. Therefore in the super resolution restoration, the condition $n\ge {r}^{2}$ must be satisfied. In our experiments, $n={r}^{2}$ is selected to obtain the only solution.

#### The number of iteration

The number of iterations is not the more the better for POCS algorithm because the adjacent points may be corrected repeatedly. The current pixel point has been corrected below the error value but the next correction may also include the current pixel point. The error value of the pixel point is improved after processing according to the correction rules. More iteration does not mean higher restoration accuracy, while the time consuming is bound to be larger. Therefore the number of iterations can be selected according to the need.

## Evaluation method of super resolution restoration based on SSIM_NCCDFT

### SSIM

SSIM (Structural SIMilarity) considers the similarity of brightness, contrast and relativity in the two images^12–14^, in which the mean value and the variance of the two images are used. SSIM is defined by:

9 $$SSIM={(l(x,y))}^{\alpha }{(c(x,y))}^{\beta }{(s(x,y))}^{\gamma }$$

where $l(x,y)$, $c(x,y)$ and $s(x,y)$ are defined as follows:

10 $$l(x,y)=\frac{2{\mu }_{x}{\mu }_{y}+{C}_{1}}{{\mu }_{x}^{2}+{\mu }_{y}^{2}+{C}_{1}}$$

11 $$c(x,y)=\frac{2{\sigma }_{x}{\sigma }_{y}+{C}_{2}}{{\sigma }_{x}^{2}+{\sigma }_{y}^{2}+{C}_{2}}$$

12 $$s(x,y)=\frac{{\sigma }_{xy}+{C}_{3}}{{\sigma }_{x}{\sigma }_{y}+{C}_{3}}$$

where *C*_1_, *C*_2_ and *C*_3_ are the structure constants. In order to simplify the operation $\alpha =\beta =\gamma =1$ is usually used. Consequently, equation (9) is changed into equation (13).

13 $$SSIM=\frac{(2{\mu }_{x}{\mu }_{y}+{C}_{1})(2{\sigma }_{xy}+{C}_{2})}{({\mu }_{x}^{2}+{\mu }_{y}^{2}+{C}_{1})({\sigma }_{x}^{2}+{\sigma }_{y}^{2}+{C}_{2})}$$

where ${\mu }_{x}$ and ${\mu }_{y}$ are the mean value of the two images and ${\sigma }_{x}^{2}$ and ${\sigma }_{y}^{2}$ are the variance of the two images. When SSIM is larger, the two images are more similar. The value range of SSIM is (0, 1).

### NCCDFT

In order to obtain the frequency domain information of the image, the Fourier transform is needed. If the discrete Fourier transform is extended to two-dimensional space, the Fourier transform to the image $f(x,y)$ of size $N\times N$ is defined by:

14 $$F(u,v)=\frac{1}{{N}^{2}}\sum _{x=0}^{N-1}\sum _{y=0}^{N-1}\,f(x,y){e}^{-j2\pi (\frac{ux+vy}{N})},u,v=0,1,{\rm{...}}\,N-1$$

**Figure 3:**
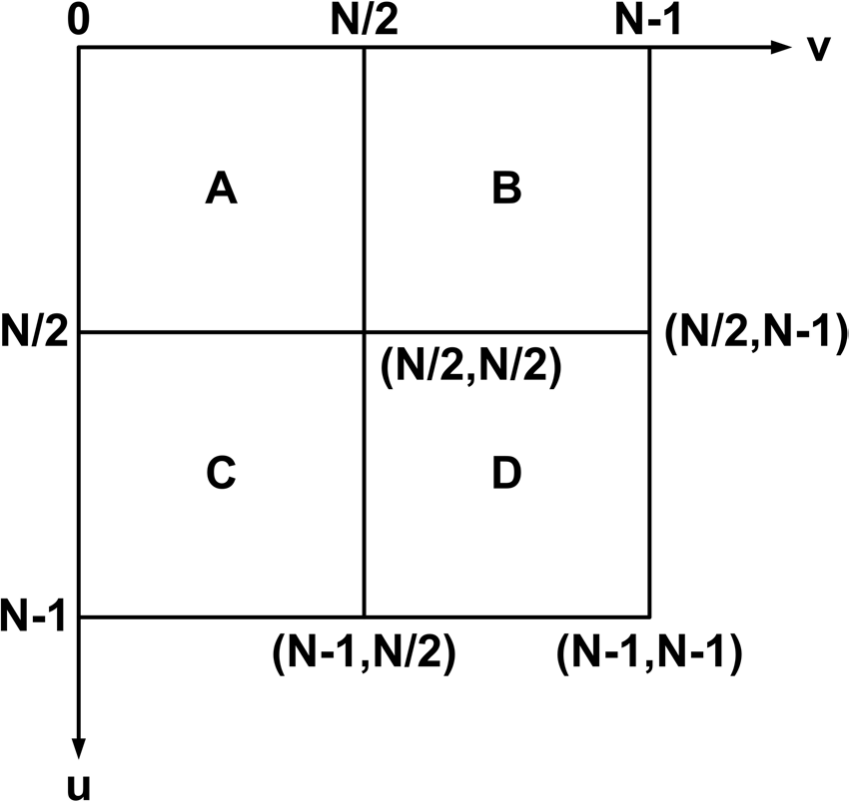
Spectrum diagram of the Fourier transforms.

The spectrum diagram of the Fourier transform is shown in Fig. 3.

The Fourier transform of the two dimensional image describes variation degree of two vertical directions in the image. The low frequency components gather at the four corners (0, 0), (0, N-1), (N-1, 0) and (N-1, N-1) as shown in the Fig. 3. The high frequency components concentrate at the centre (N/2, N/2). The most energy of the image concentrates on low frequency components. Consequently, the corners are bright and the centre is dark in the figure of Fourier transform, which is not good for analyzing the image. Therefore, we transform it by utilizing the periodicity and conjugate symmetry of Fourier transform to make the low frequency components to be gathered at the centre and the high frequency components to be gathered at the corners.

In the super resolution image restoration, we assume origin high resolution image is $g(x,y)$, the size is $N\times N$ and its Fourier transform is $G(u,v)$. The high resolution restored image $\hat{g}(x,y)$ is estimated from $g(x,y)$, which Fourier transform is $\hat{G}(u,v)$. The better the effect of restoration is, the closer between $\hat{g}(x,y)$ and $g(x,y)$ is, and the closer between $\hat{G}(u,v)$ and $G(u,v)$ is. Therefore the concept of Normalized Cross Correlation of DFT is proposed in this paper. NCCDFT uses the similarity degree of Fourier transform to measure the effect of super resolution restoration. NCCDFT is defined by:

15 $$NCCDFT(g(x,y),\hat{g}(x,y))=\frac{\sum _{u=0}^{N-1}\sum _{v=0}^{N-1}(G(u,v)\cdot \hat{G}(u,v))}{\sqrt{\sum _{u=0}^{N-1}\sum _{v=0}^{N-1}G{(u,v)}^{2}\cdot \sum _{u=0}^{N-1}\sum _{v=0}^{N-1}\hat{G}{(u,v)}^{2}}}$$

Where $G(u,v)$ and $\hat{G}(u,v)$ are the Fourier transform of $g(x,y)$ and $\hat{g}(x,y)$, and $NCCDFT\in (0,1]$. The larger the NCCDFT, the more similar the Fourier transforms is, and the better the quality of super resolution restoration is.

### SSIM_NCCDFT

In the above section, NCCDFT evaluates the quality of restoration image in the frequency domain. Meanwhile the restoration high resolution image $\hat{g}(x,y)$ and the original high resolution image are similar in the spatial domain if the restoration quality is good enough. There are many evaluation method of image similarity in the spatial domain. SSIM measures the characteristics of image from three aspects: brightness, contrast and relativity, which is comprehensive and direct. Consequently SSIM is chosen to measure the similarity of super resolution restoration image in the spatial domain by:

16 $$SSIM(g(x,y),\hat{g}(x,y))=\frac{(2{\mu }_{g}{\mu }_{\hat{g}}+{C}_{1})(2{\sigma }_{g\hat{g}}+{C}_{2})}{({\mu }_{g}^{2}+{\mu }_{\hat{g}}^{2}+{C}_{1})({\sigma }_{g}^{2}+{\sigma }_{\hat{g}}^{2}+{C}_{2})}$$

Where *μ* and *σ* are mean value and variance of the image respectively.

In order to combine the spatial domain and the frequency domain, this paper proposes an evaluation method of super resolution restoration based on SSIM_NCCDFT as:

17 $$SSIM\text{\_}NCCDFT(g,\hat{g})=SSIM{(g,\hat{g})}^{\alpha }NCCDFT{(g,\hat{g})}^{\beta }$$

where *α* and *β* are the weighted factor of spatial domain and the weighted factor of frequency domain respectively, which are used to adjust the proportion of evaluation index between the spatial domain and frequency domain. Generally speaking $\alpha =\beta =0.5$.

Evaluation method of super resolution restoration based on SSIM_NCCDFT measures the similarity degree of the statistical characteristics in spatial domain between the restoration image and the original high resolution image. Meanwhile it also considers the recovery degree of the restored image in the frequency domain. It comprehensively evaluates the effect of the super resolution restoration image. It has a certain guiding significance for the quality evaluation system of the super resolution restoration image.

## Experimental evaluations of BPOCS

### Generation of image degradation sequence

In the super resolution restoration algorithm, the used image sequences can be several continuous frames taken by the imaging system or generated by down sampling from high resolution image. So far the common evaluation methods of super resolution restoration algorithms such as PSNR (Peak Signal to Noise Ratio), SSIM and SSIM_NCCDFT need the original high resolution image. The original high resolution image is hard to obtain if the image sequences are several continuous frames taken by the imaging system. Therefore, the low resolution images are generated by program processing in the evaluation experiment.

Firstly the process of noise and blurring are needed to simulate. The image signal will be disturbed by various noises and affected by blurring before reaching the target surface of the imaging system. The actual noise can be approximated as Gauss white noise. The relative motion of the target and imaging system that is caused by camera shaking can be approximated as motion blurring of one certain direction. Therefore the Gauss white noise with variance *σ* and the motion blurring of one certain direction with length $len$ are added into the visible light high resolution Lena image, Barbara image, infrared high resolution missile image, and the plane image. In the experiment, $\sigma =0.01$ and $len=5$.

The contaminated image signal reaches the target surface of the imaging system. The imaging process of the camera is a process of down sampling. Therefore the original high resolution images need to be down sampled.

**Figure 4:**
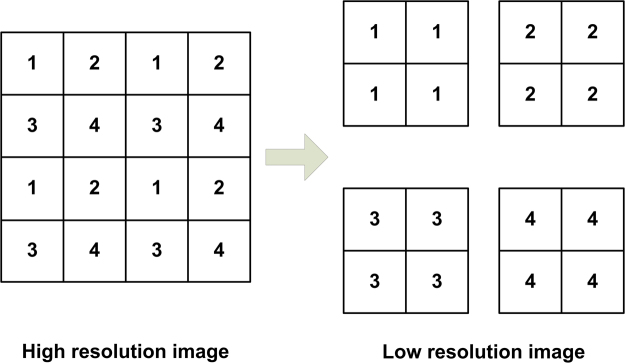
Down-sampling process.

The down-sampling process is shown in Fig. 4.

From the experimental results in Tables 1 and 2, we can see that the proposed method has better performance than bilinear interpolation and traditional POCS.

One frame of high resolution image can generate four frames low resolution images using the method of interlace row and column sampling. Another way to generate four frames of low resolution images is to randomly select one of the four adjacent pixels.

In summary, the steps which generate low resolution images from the high resolution image are as follows.

Step1: The white Gaussian noise with variance $\sigma$ and the motion blurring of a certain direction with length $len$ are added to the original high resolution image.

Step2: The low resolution image sequences are obtained by down-sampling from the contaminated high resolution image.

**Figure 5:**
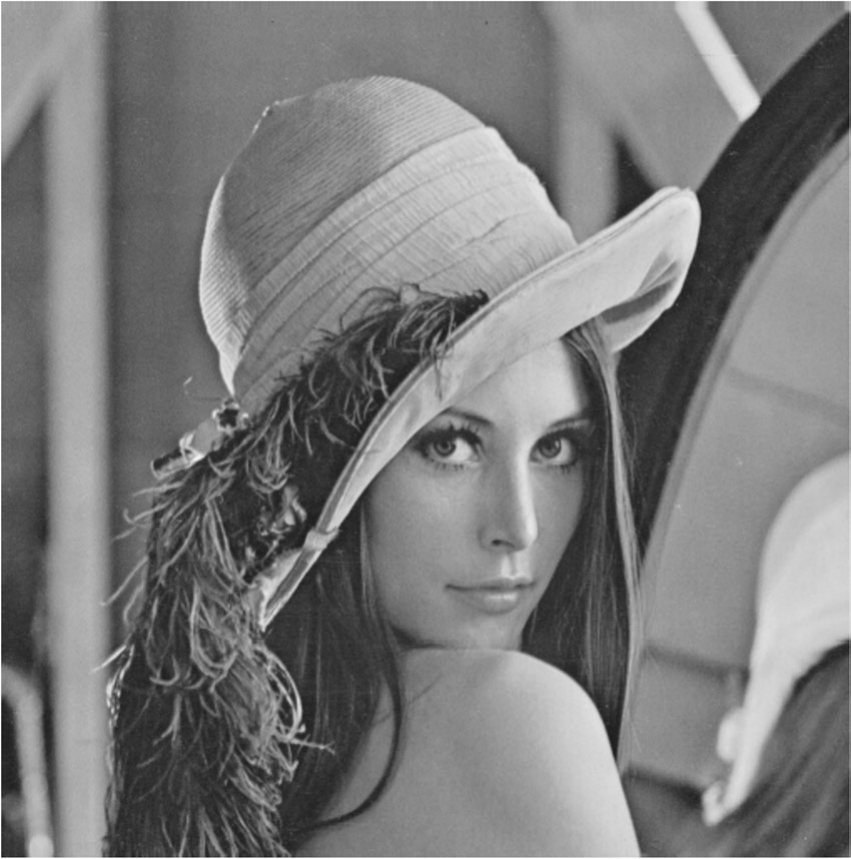
Lena image.

The Lena image with resolution 512×512 is shown in Fig. 5.

The visible light Lena image finally generates the low resolution image sequence using the above processing.

**Figure 6:**
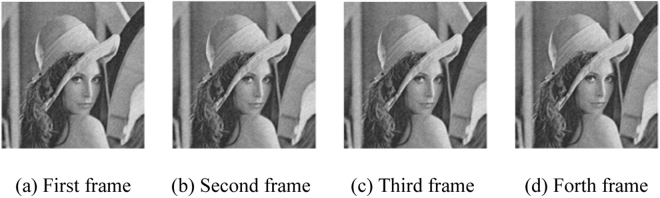
Low resolution image sequence of down-sampled Lena image.

The resolution of Lena image after down-sampling, as shown in Fig. 6, is 256×256.

**Figure 7:**
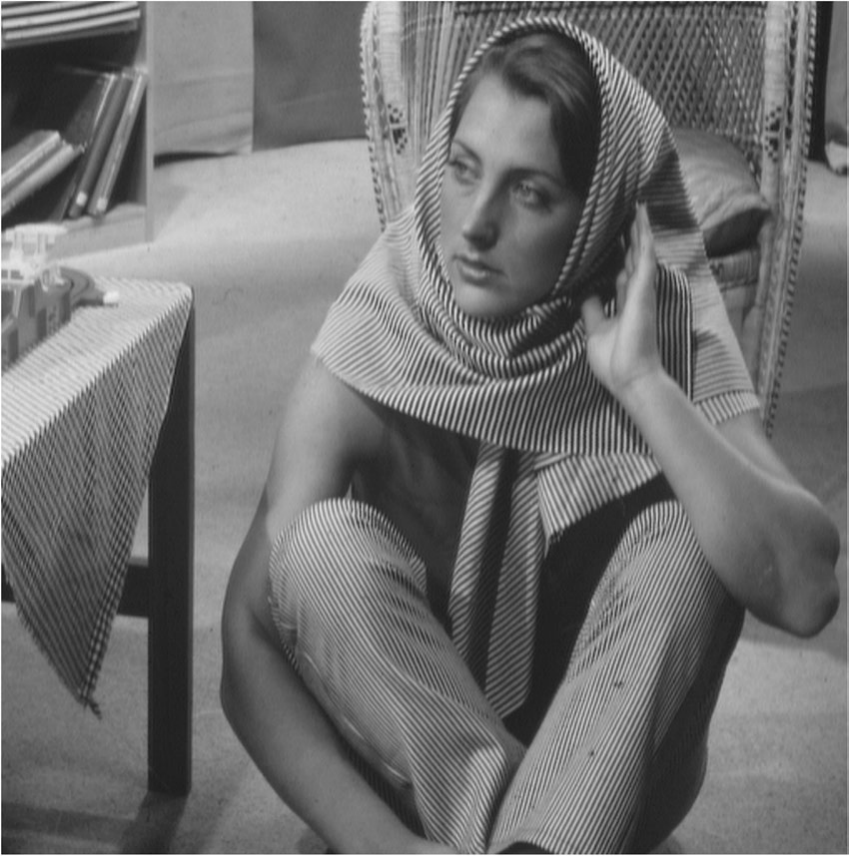
Barbara image.

The Barbara image with resolution 512×512 is shown in Fig. 7.

The visible light image Barbara finally generates the low resolution image sequence using the above processing.

**Figure 8:**
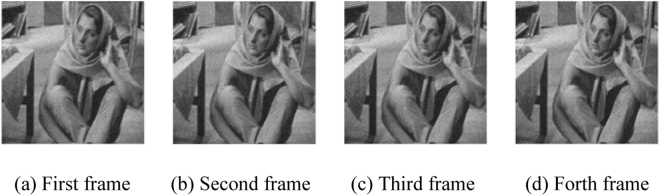
Low resolution image sequence of down-sampled image Barbara.

The resolution of Barbara image after down-sampling, as shown in Fig. 8, is 256×256.

**Figure 9:**
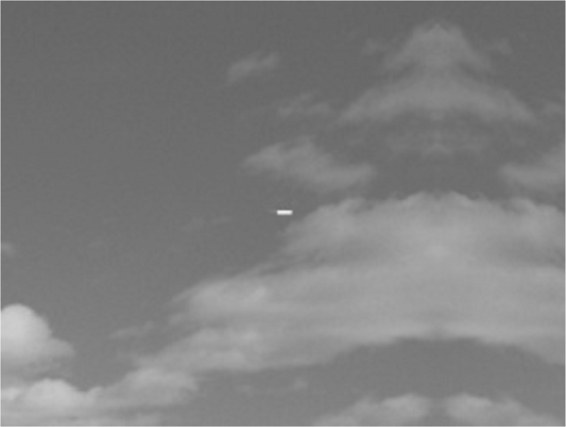
Missile image.

The Missile image with resolution 320 × 240 is shown in Fig. 9.

The infrared missile image finally generates the low resolution image sequence using the above processing.

**Figure 10:**
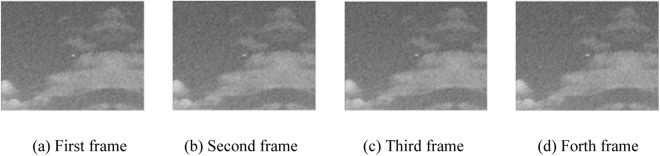
Low resolution image sequence of missile image after down-sampling.

The resolution of missile image after down-sampling, as shown in Fig. 10, is 160 × 120.

**Figure 11:**
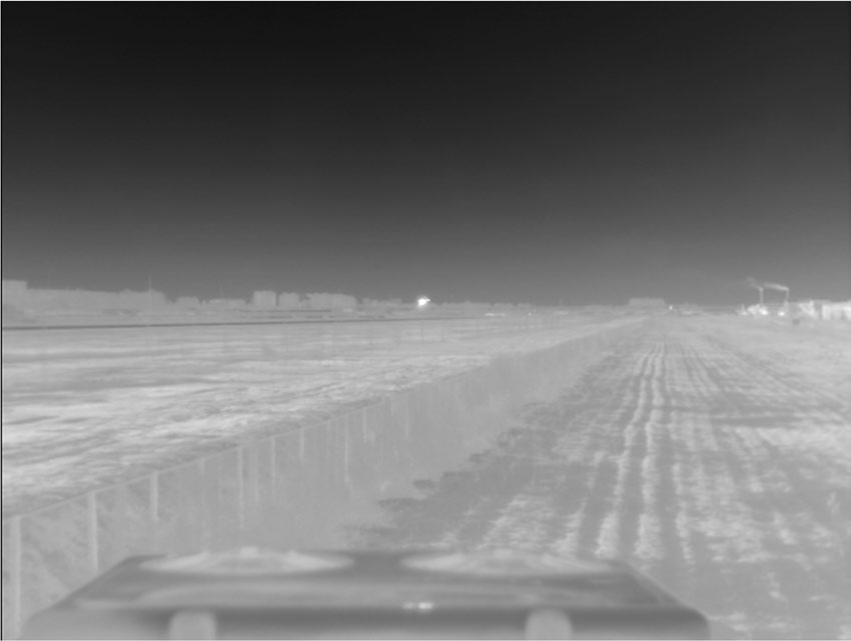
Plane image.

The Plane image with resolution 320 × 240 is shown in Fig. 11.

The infrared plane image finally generates the low resolution image sequence using the above processing.

**Figure 12:**
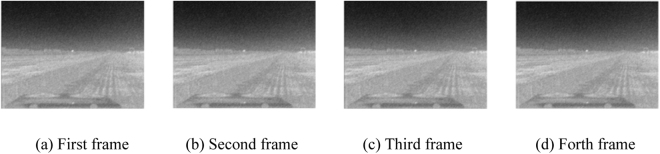
Low resolution image sequence of plane image after down-sampling.

The resolution of plane image after down-sampling, as shown in Fig. 12, is 160 × 120.

### Experimental results and analysis

The low resolution image sequences generated above are restored by bilinear interpolation restoration, traditional POCS restoration, and the proposed POCS super resolution restoration based on BM3D. The restoration performance is evaluated by SSIM_NCCDFT. The iteration number is 10. POCS super resolution restoration arithmetic based on BM3D takes ${\tau }_{m}=0.1$ and ${\tau }_{N}=15$.

**Figure 13:**
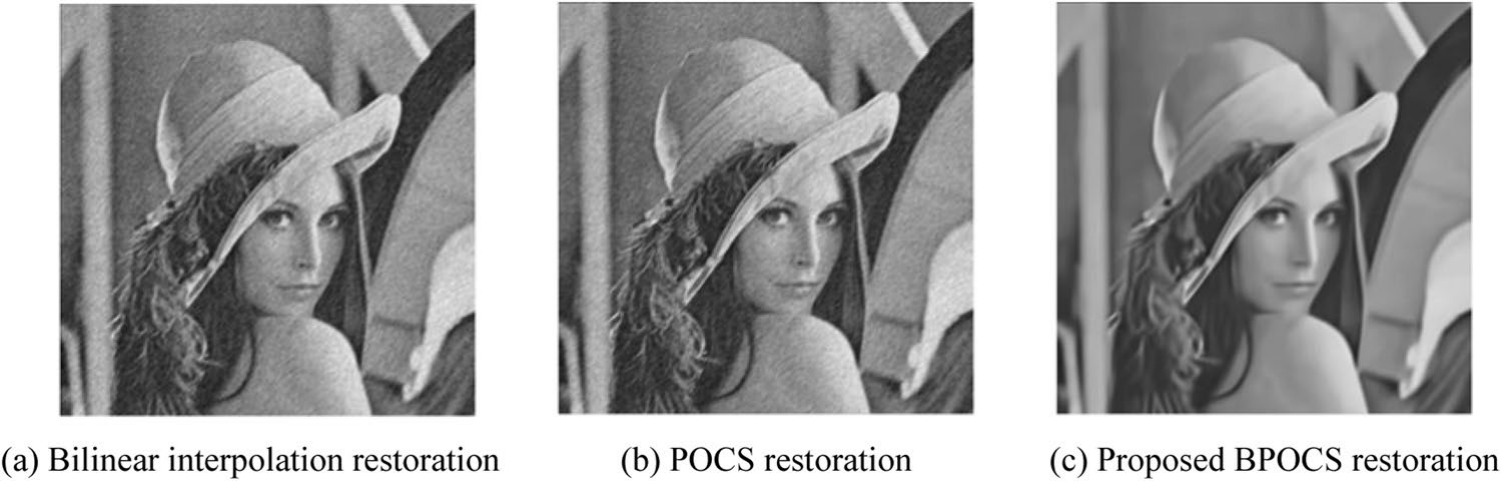
Restoration effect of Lena image.

The resolution of restored Lena image is 512 × 512. The restoration effect of Lena image is shown in Fig. 13.

**Figure 14:**
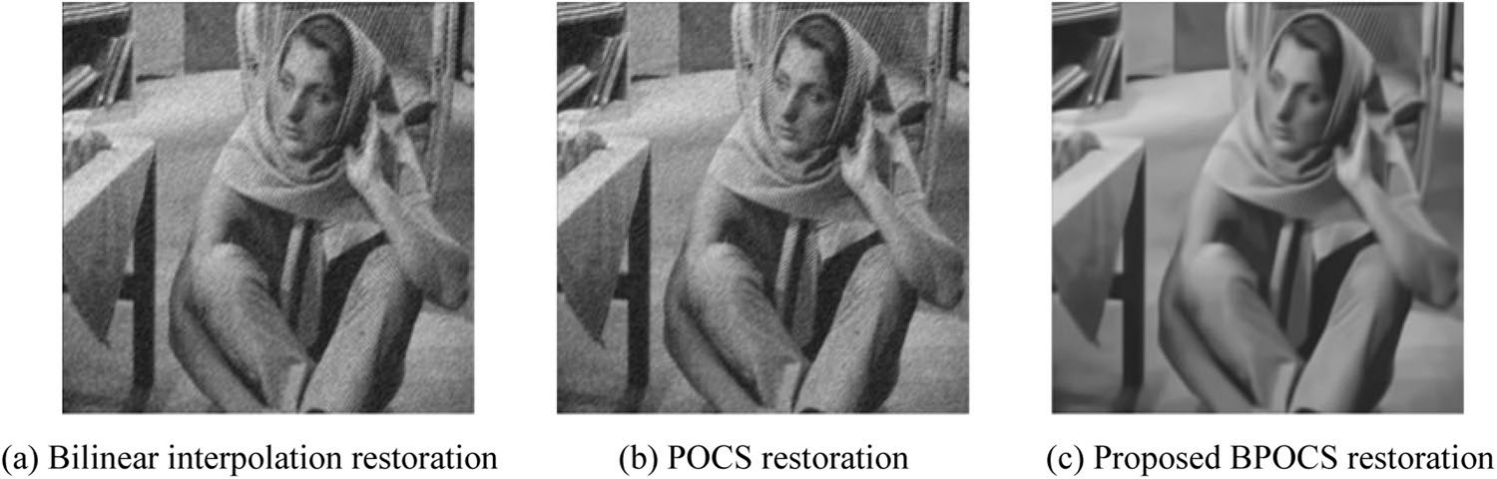
Restoration effect of Barbara image.

The resolution of restored Barbara image is 512 × 512. The restoration effect of Barbara image is shown in Fig. 14.

**Figure 15:**
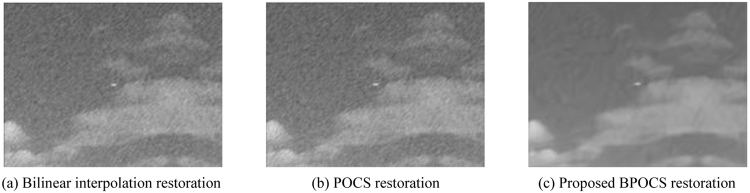
Restoration effect of missile image.

The resolution of restored missile image is 320 × 240. The restoration effect of missile image is shown in Fig. 15.

**Figure 16:**
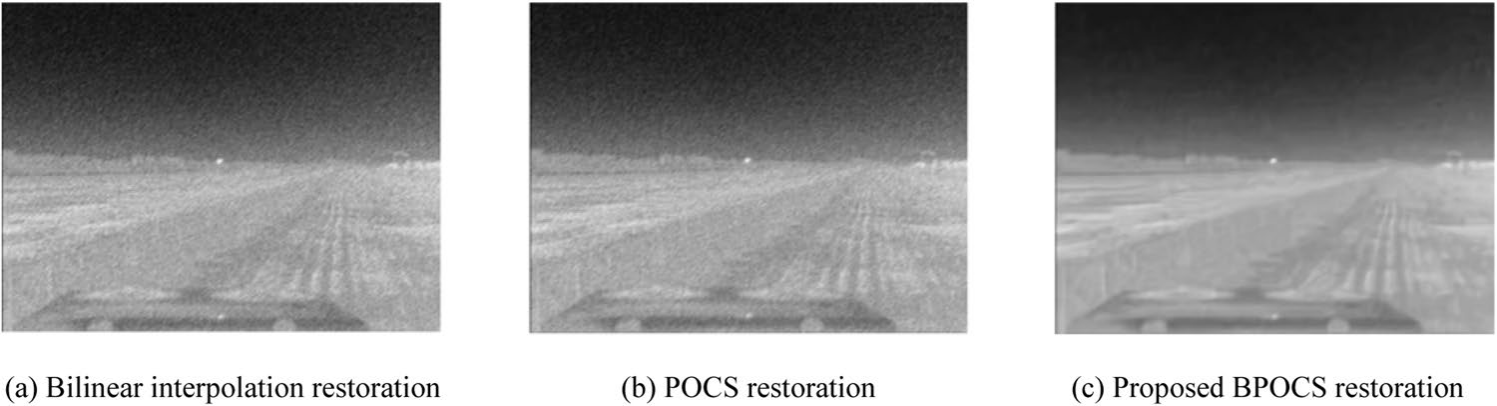
Restoration effect of plane image.

The resolution of restored plane image is 320 × 240. The restoration effect of plane image is shown in Fig. 16.

From the subjective effect of the restored images from Figs 13 to 16 we can see that the restored images using bilinear interpolation and traditional POCS appear with noise and blurring, while the restored image using the proposed POCS super resolution restoration based on BM3D (BPOCS) is clear. There is no noise which can be perceived from subjective observation. The restored image contains information from multiple frames. Consequently, it has larger target size after mapping to the high resolution grid. The edge is clearer than that of using bilinear interpolation method.

The three restoration algorithms are evaluated by PSNR and SSIM_NCCDFT.

The PSNR evaluation contrast of the three restoration algorithm is shown in Table 1.

**Table 1:** PSNR evaluation contrast of the three restoration algorithms.

Image	Bilinear (dB)	POCS (dB)	BPOCS (dB)
**Lena**	26.75	27.53	27.96
**Barbara**	23.06	23.94	24.27
**Missile**	27.03	29.24	32.18
**Plane**	28.01	29.97	33.34

**Table 2:** SSIM_NCCDFT evaluation contrast of the three restoration algorithms.

Image	Bilinear (dB)	POCS (dB)	BPOCS (dB)
**Lena**	0.9736	0.9870	0.9920
**Barbara**	0.9612	0.9671	0.9725
**Missile**	0.9426	0.9684	0.9813
**Plane**	0.9713	0.9825	0.9912

PSNR is defined by equation 18:

18 $$PSNR=10\times \,{\rm{lg}}({({2}^{n}-1)}^{2}/MSE)$$

The SSIM_NCCDFT evaluation contrast of the three restoration algorithms is shown in Table 2. SSIM_NCCDFT is defined by equation 17.

### Analysis of experimental results

The traditional POCS restoration method has poor ability to depress noise and maintain edge detail and its processing time is longer, which is not suitable to be applied to the photoelectric tracking system.

The proposed method has strong ability to depress noise. From the restoration results, the noise of background is filtered out. The overall restoration performance is good. It takes better restoration effect than traditional POCS restoration method. The noise in the high resolution restored image cannot be perceived from subjective observation.

## Conclusions

Aiming at solving the problem of traditional POCS restoration method, which is sensitive to noise, this paper combines the POCS restoration and BM3D filtering method together. Firstly the low resolution image sequences are filtered by BM3D. Secondly the registration and estimation of motion parameters are performed on the filtered image sequences. Finally the low resolution image sequences are mapped to the high resolution grid for correction and restoration. Meanwhile, the BM3D method is optimized to reduce the computation complexity using pre-filtering mean value of image blocks and limiting the number of image blocks. Experimental results show that the proposed method has better restoration performance than traditional POCS method when the low resolution images have noise. The restored high resolution image appear no noise from subjective observation.

## References

[1] Zhou L, Liu F, Zhu XC (2006). A novel method to realize compressed video super-resolution reconstruction. J of Electron..

[2] Varghese J, Tairan N, Subash S (2015). Adaptive switching non-local filter for the restoration of salt and pepper impulse-corrupted digital images. Arab J Sci Eng..

[3] Shreyamsha Kumar BK (2013). Image denoising based on non-local means filter and its method noise thresholding. SIViP..

[4] Matsuoka J, Koga T, Suetake N, Uchino E (2016). Switching non-local vector median filter. Opt Rev..

[5] Gao JJ, Chen XH, Li JY, Liu GC, Ma J (2010). Irregular seismic data reconstruction based on exponential threshold model of POCS method. Appl Geophys..

[6] Tian B, Sclabassi RJ, Hsu JT, Liu Q, Li CC (2007). Sun, M.G. A wavelet transform based POCS super resolution algorithm. J of Electron..

[7] Ge ZJ, Li JY, Pan SL, Chen XH (2015). A fast-convergence POCS seismic denoising and reconstruction method. Appl Geophys..

[8] Djurovic I (2016). BM3D filter in salt-and-pepper noise removal. J Image Video Proc..

[9] Zhang H, Liu WJ, Wang RL, Liu T, Rong MT (2016). Hardware architecture design of block-matching and 3D-fitering denoising algorithm. J Shanghai Jiaotong Univ Sci..

[10] Eksioglu EM (2016). Decoupled algorithm for MRI reconstruction using nonlocal block matching model: BM3D-MRI. J Math Imaging Vis..

[11] Wen DH, Jiang YS, Zhang YZ, Gao Q (2014). Statistical properties of polarization image and despeckling method by multiresolution block-matching 3D filter. Opt Spectrosc..

[12] Rehman A, Rostami M, Wang Z, Brunet D, Vrscay ER (2012). SSIM-inspired image restoration using sparse representation. EURASIP J Adv Signal Process..

[13] Peng Q, Zhang L, Wu X, Wang QH (2014). Modeling of SSIM-based end-to-end distortion for error-resilient video coding. J Image Video Proc..

[14] Huang Y, Chen X, Ding XH (2016). A harmonic means pooling strategy for structural similarity index measurement in image quality assessment. Multimed Tools Appl..

